# Decrease in Neutrophil-to-Lymphocyte Ratio during Neoadjuvant Chemotherapy as a Predictive and Prognostic Marker in Advanced Ovarian Cancer

**DOI:** 10.3390/diagnostics11071298

**Published:** 2021-07-20

**Authors:** Elisabetta Sanna, Luciana Tanca, Cristina Cherchi, Giulia Gramignano, Sara Oppi, Maria Gloria Chiai, Antonio Macciò, Clelia Madeddu

**Affiliations:** 1 Department of Gynecologic Oncology, A. Businco Hospital, ARNAS G. Brotzu, 09100 Cagliari, Italy; dr.elisabettasanna@gmail.com (E.S.); mariagloria.chiai@ostetricheca.postecert.it (M.G.C.); 2 Department of Medical Oncology, A. Businco Hospital, ARNAS G. Brotzu, 09100 Cagliari, Italy; l.tanca@virgilio.it (L.T.); cristina.cherchi@aob.it (C.C.); 3 Medical Oncology Unit, “Nostra Signora di Bonaria” Hospital, 09037 San Gavino, Italy; giuli.gramignano@gmail.com; 4 Hematology and Transplant Center, A. Businco Hospital, ARNAS G. Brotzu, 09100 Cagliari, Italy; sara.oppi@gmail.com; 5 Department of Medical Sciences and Public Health, University of Cagliari, 09100 Cagliari, Italy; clelia_md@yahoo.it

**Keywords:** ovarian cancer, neoadjuvant chemotherapy, neutrophil-to-lymphocyte ratio, inflammation, c-reactive protein, interleukin-6, progression-free survival, Glasgow prognostic score

## Abstract

Since chronic inflammation is associated with ovarian cancer growth and progression, some clinical studies have assessed the association between the pre-treatment neutrophil-to-lymphocyte ratio (NLR) and the prognosis of ovarian cancer. The purpose of this study was to assess the dynamic behavior of the NLR during the course of neoadjuvant chemotherapy (NACT) in patients with high grade serous (HGS) advanced epithelial ovarian cancer and assess its correlation with clinical response, progression free survival (PFS) and changes in other inflammatory indexes. We performed a prospective observational study on 161 patients who underwent NACT at the Department of Gynecologic Oncology, ARNAS G. Brotzu, Cagliari, between 2009 and 2019. NLR was evaluated before starting and after three cycles of NACT. Based on response after three cycles of NACT, patients were divided into two groups: responsive and non-responsive. The primary endpoint was to assess the predictive role of NLR by comparing the responsive and non-responsive patients at baseline and after three cycles of NACT. Secondary endpoints were (a) to correlate NLR with other inflammation markers (CRP, fibrinogen, ferritin, IL-6), albumin, and modified Glasgow Prognostic Score (mGPS) with NLR at baseline and after NACT; (b) to assess the association between NLR and PFS. We found that the NLR value at baseline was not associated with response to NACT, while a decrease in NLR after three cycles was correlated with a better response to NACT. Also, values of CRP, IL-6, ferritin, and mGPS after three cycles of NACT (but not at baseline) were significantly associated with clinical response. Moreover, we found that patients with a low NLR value after 3 cycles of NACT, but not at baseline, had a significantly higher PFS than patients with high NLR after 3 cycles of NACT. In conclusion, NLR change during treatment could serve as a predictive marker of response to NACT in patients with HGS advanced ovarian cancer. This allows for the early identification of non-responsive patients who will need treatment remodeling.

## 1. Introduction

The link between inflammation and cancer has been well-established since 1863 when Rudolf Virchow observed leukocytes infiltrates in neoplastic tissue and hypothesized that there was an association between the origin of malignancies and sites of chronic inflammation [1]. Leukocyte infiltrates, present in almost all tumors, can be found in different locations and have different cellular components. They include neutrophils, dendritic cells, macrophages, eosinophils, mast cells, and lymphocytes. During the early stages of neoplastic initiation, the immune system controls oncogenesis, while in the further phases it has been hypothesized that it can promote tumor growth and metastasis [2,3]. Initially, T lymphocytes, dendritic cells, and natural killer cells can limit cancer development (phase of resistance), but an immunosuppressive tumor microenvironment (TME) can override these effects over time, thus promoting cancer development and metastases [4]. Therefore, neoplastic progression is associated with immunosuppression. In fact, as the tumor expands and the TME changes, the ability of the immune system to activate T cells and direct them toward the tumor is modified [2,5]. Thus, the growth of a tumor that can overcome the resistance mechanisms indicates a lack of efficacy of the specific immune response and cancer hyper-aggressivity [6,7]. Immunopathology and necrosis are associated with this condition, and they elicit a local non-specific inflammation that extends to the systemic level and is responsible for symptoms and metabolic modifications, which modify the overall clinical state of a patient [8]. This is known as the “tolerance phase” and it mostly occurs to limit the damages related to the failure of the resistance phase [9]. In this phase of tolerance, the role of macrophages is central, and the events related to their activation, such as the associated cytokine storm, presence of elevated reactive oxygen species (ROS), dysregulated metabolism of iron, and modified glucose metabolism (Warburg effect) induce an unfavorable metabolic environment that impairs the functions of T-cells and their anti-tumoral activity [8,10,11,12]. Worsening lymphopenia reflects the altered lymphocytic functions at the systemic level [13]. As the process of inflammation is associated with cancer growth, several systemic inflammatory markers have been studied for their possible role as predictors of clinical outcomes and prognosis of several cancer types [14]. The neutrophil-to-lymphocyte ratio (NLR) has been proposed as one of the most helpful prognostic indexes [15,16]. It is calculated from the ratio between the neutrophil and lymphocyte counts from a full blood count. Recently, some clinical studies have revealed the association between pre-treatment NLR and the prognosis of ovarian cancer [17,18,19,20,21,22,23,24,25,26,27,28,29]. Convinced that this condition can be modified by antineoplastic treatments, we assessed the behavior of NLR during the course of chemotherapy in patients with advanced ovarian cancer who received neoadjuvant chemotherapy (NACT) and studied its correlation with clinical response, survival and the primary inflammatory indexes.

## 2. Materials and Methods

We conducted a prospective observational study and enrolled consecutive patients with ovarian, fallopian-tube, or primary peritoneal high grade serous carcinoma (HGSC), who were classified according to the International Federation of Gynecology and Obstetrics (FIGO) as patients with stage III/IV cancers. These patients were referred to our center between June 2009 and December 2019 and were NACT candidates. This study was approved by the local independent Institutional Ethics Committee of the Azienda Ospedaliero Universitaria, in Cagliari, Italy, and was carried out in accordance with the principles of the Declaration of Helsinki. All enrolled patients provided written informed consent for participation in the study and for the use of their biological samples for laboratory analyses.

### 2.1. Inclusion and Exclusion Criteria

Patients were included in the study based on the histological findings of the above-mentioned cancers, i.e., high grade serous ovarian, fallopian-tube or primary peritoneal carcinoma, advanced stage (III-IV), and indication for NACT treatment with a platinum-based regimen. The chemotherapy protocol was administered according to a weekly schedule. Exclusion criteria included other hystotypes of ovarian cancer (i.e., mucinous, endometrioid, clear cell), and ineligibility for NACT due to comorbidities or incomplete NACT cycles. Eligibility for NACT was based on each patient’s clinical features, including extent of disease, presence/absence of a comorbidity, performance status, imaging techniques’ reports (trans-vaginal/trans-abdominal ultrasonography, computed tomography [CT], and positron emission tomography-computed tomography [PET/CT]). Eligibility was also determined by a direct laparoscopic visualization of the pelvic and abdominal cavities, which evaluated the possibility of resection; when there was a low probability to perform an optimal surgical cytoreduction (residual disease ≤1 cm), patients were considered eligible for NACT. In addition, patients were considered as NACT candidates if they had large abdominal and pelvic spread of the disease (unresectable massive peritoneal and diaphragmatic involvement, mesenterial retraction, miliary carcinomatosis of the bowel, liver, and stomach metastases) [30].

### 2.2. Measures and Outcomes

NLR was calculated as the neutrophil percentage value divided by the lymphocyte percentage value. We analyzed NLR at baseline and after three cycles of standard platinum-based chemotherapy. All cancers were classified at diagnosis in accordance with FIGO staging. Objective tumor response to treatment was assessed according to the Response Evaluation Criteria in Solid Tumors (RECIST) criteria with CT and/or PET-CT scans, together with cancer antigen 125 (CA125) and human epididymis protein 4 (HE4), at baseline and after three cycles of NACT. Based on response after three cycles of NACT, patients were divided into two groups: the responsive (group 1) and non-responsive (group 2) group. Furthermore, responsive patients underwent interval debulking surgery. 

In patients who underwent interval debulking surgery, chemotherapy response scores (CRS) were assigned to omental and adnexal metastases, and categorized as no/minimal (CRS1), partial (CRS2), and complete/near-complete (CRS3) response to neoadjuvant chemotherapy according to Bohm et al. [31]. In brief, CRS1 corresponds to no or minimal tumor response (no or minimal regression-associated fibroinflammatory changes limited to a few foci), cases in which it is difficult to decide between regression and tumor-associated desmoplasia or inflammatory cell infiltration; CRS2 means appreciable tumor response with viable tumor readily identifiable, ranging from multifocal or diffuse regression-associated fibroinflammatory changes with viable tumor in sheets, streaks, or nodules to extensive regression-associated fibroinflammatory changes with multifocal residual tumors; and CRS3 corresponds to complete absence of tumor cells or individual cells, cell groups, or nodules with maximum size of 2 mm [32].

Progression-free survival (PFS) was defined as the time from randomization to objective disease progression based on imaging (according to RECIST criteria). To evaluate the severity of inflammation, we assessed the levels of C-reactive protein (CRP), fibrinogen, ferritin, interleukin (IL)-6 at baseline and after three cycles of chemotherapy. Moreover, we assessed the value of the modified Glasgow prognostic score (mGPS), an inflammatory/nutritional index that assesses the correlation between inflammation (CRP) and nutritional status (albumin levels). This index was interpreted as follows: 2, elevated CRP (≥10 mg/L) and low albumin (<3.5 g/dL); 1, elevated CRP only; and 0, normal CRP (<10 mg/L) [33].

### 2.3. Data Analysis

We evaluated the following parameters for each patient: patient’s general features (age, weight, height, and body mass index), documented comorbidities, FIGO stage, histologic type, number of NACT cycles, type of chemotherapy and objective tumor response after NACT, PFS, pre-operative markers (CA125 and HE4), hematological parameters (NLR), markers of inflammation (CRP, fibrinogen, ferritin, and IL-6), albumin, and the mGPS score. Blood cell count, CRP, fibrinogen, ferritin, and albumin were assessed via routine laboratory assays. Serum levels of IL-6 were assessed via an enzyme-linked immunosorbent assay according to previously described procedures in our previous studies [34]. 

### 2.4. Endpoints of the Study

The primary endpoint was the assessment of the predictive role of NLR in determining patient outcome by comparing NLR values between the responsive and non-responsive patients at baseline and after three cycles of NACT. The secondary endpoint was the comparison of inflammation markers, and mGPS between the two groups, and their correlation with NLR at baseline and after NACT. We evaluated also the correlation between NLR and tumor markers CA125 and HE4, as well as the association between NLR and CRS. Among secondary endpoints, we also assessed the correlation between NLR and PFS.

### 2.5. Statistical Analysis

Based on our preliminary data, we anticipated a mean difference of 1.6 between groups and an expected standard deviation (SD) of 2. Using this calculation and considering an α-type error of 0.05 and β-type error of 0.10, we planned to enroll at least 152 patients. Data were reported as the mean ± SD. Differences between means were examined by a two-tailed, unpaired t-test for normally distributed variables or by the Mann–Whitney test for non-normally distributed variables. After checking the linearity of the data distribution and the variability among the groups, the differences in the mean levels of NLR, and other laboratory variables at baseline and after three cycles of NACT were compared across groups by ANOVA for repeated measures. The Pearson test (or Spearman for non-parametric variables) was used to assess the correlation between variables. To assess the predictive role of NLR in the detection of the objective tumor response, we used the receiver operating characteristic (ROC) curve analysis and the respective area under the curve (AUC) to determine the sensitivity and specificity of the NLR. The AUC of ROC analyses using NLR values showed values higher than 0.80, and therefore, ROC analyses were used to determine the appropriate threshold value of this variable. Then, survival analyses were performed after the patients were divided into 2 groups according to the NLR cut off value using Kaplan–Meier curves and log-rank analysis to compare PFS between groups. HR and CI were calculated from univariate Cox regression survival analysis. All reported *p*-values are two-tailed, and *p* < 0.05 was considered statistically significant. All statistical analyses were performed using MedCalc Software version 19.6 (MedCalc Software Ltd., Ostend, Belgium).

## 3. Results

From June 2009 to December 2019, 161 patients were enrolled in the study. The median age of the patients at the time of diagnosis was 57 years (range: 39–86). The clinical characteristics and laboratory parameters at baseline are reported in Table 1 and Table 2, respectively. Notably, the assessment of CRP and fibrinogen showed that 147 patients (91%) had inflammation. In addition, after three cycles of platinum-based chemotherapy, 132 (82%) patients were responsive, whereas 29 (18%) were non-responsive. Distribution of patients assigned to each of the three CRS groups was as follows: 23.2% patients were categorized as CRS1, 46.8% patients as CRS2, and 30% patients as CRS3.

One-hundred forty-seven patients were assessable for PFS; median PFS was 14 (range: 4–30) months.

### 3.1. Primary Endpoints: Comparison of NLR at Baseline and after Three Cycles of NACT between Groups 1 and 2

The mean value of baseline NLR was not significantly different (4.8 ± 2.9 in group 1 versus 7.3 ± 6.7 in group 2, *p* = 0.266) between the two groups. However, the mean value of NLR after three cycles of NACT was significantly different (1.2 ± 0.8 in group 1 versus 2.4 ± 0.5 in group 2, *p* < 0.0001) between the two groups.

**Figure 1:**
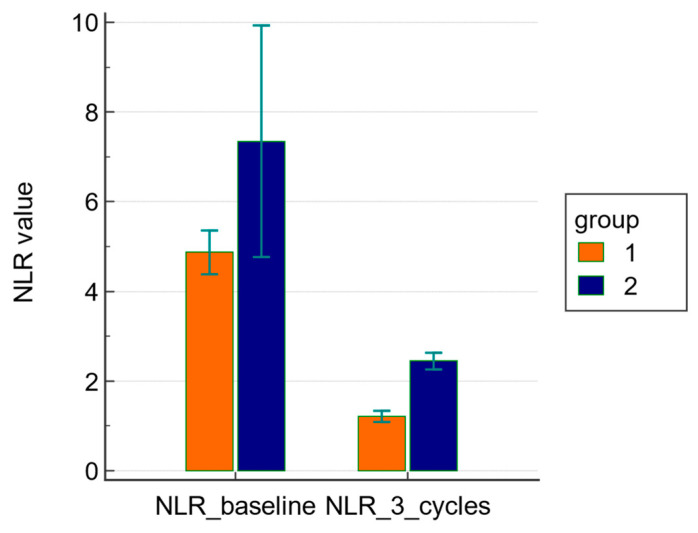
ANOVA for repeated measures showed a significant difference of the mean changes of NLR at baseline and after three cycles of NACT.

ANOVA for repeated measures showed a significant difference (*p* < 0.001) in the mean changes of NLR before and after treatment between the two groups; the decrease was significantly higher in group 1 than in group 2 (Figure 1).

### 3.2. Secondary Endpoints

Correlation analysis demonstrated a positive association between baseline NLR and CRP values (R = 0.469, *p* < 0.0001; 95% CI: 0.336 to 0.584) as well as between NLR and CRP values after the three treatment cycles (R = 0.422, *p* < 0.0001; 95% CI: 0.286 to 0.541; Table 3). Fibrinogen was not significantly correlated with NLR either at baseline or after the three NACT cycles. Significant correlations were found between baseline NLR and IL-6, ferritin, and mGPS. Additionally, significant correlations between NLR and IL-6, ferritin, and mGPS were observed after NACT (Table 3). 

As regards the correlation between NLR and CA124 and HE4, we found a significant positive correlation between NLR after three cycles of chemotherapy and CA125 (r = 0.7904, *p* = 0.0013, 95% CI: 0.4241 to 0.9344) and HE4 (r = 0.7688, *p* = 0.0035, 95%CI: 0.3488 to 0.9317) after three cycles of chemotherapy, while no significant correlation was found at baseline.

#### 3.2.1. Comparison of CRP, Fibrinogen, IL-6, Ferritin, Albumin, and mGPS Levels at Baseline and after Three Cycles of NACT between Groups 1 and 2

The mean value of CRP was not significantly different between the two groups (5.6 ± 5.8 in group 1 versus 5.9 ± 6.6 in group 2, *p* = 0.9084) at baseline, but was significantly different between the two groups after treatment (0.4 ± 0.3 in group 1 versus 1.3 ± 1.8 in group 2, *p* = 0.0038; Table 4). The mean value of fibrinogen was not significantly different between the two groups (556 ± 207 in group 1 versus 598 ± 217 in group 2, *p* = 0.1893) at baseline, as well as after three NACT cycles (373 ± 88 in group 1 versus 400 ± 114 in group 2, *p* = 0.1894; Table 4). The mean values of IL-6, ferritin, and mGPS were not significantly different between the two groups at baseline (Table 2), whilst they were significantly different after NACT (Table 4). 

#### 3.2.2. Correlation Analysis between NLR, CPR, Fibrinogen, IL-6, Albumin, mGPS, and Objective Tumor Response

Correlation analysis by the Spearman test showed a significant association between objective tumor response and NLR (R = 0.554; 95% CI: 0.437 to 0.653, *p* < 0.0001), CRP (R = 0.4549; 95% CI: 0.3163 to 0.5745; *p* < 0.0001), IL-6 (R = 0.254; 95% CI: 0.0554 to 0.3646; *p* = 0.0088), ferritin (R = 0.210; 95% CI: 0.0498 to 0.3597; *p* = 0.0107), and mGPS (R = 0.229; 95% CI: 0.0765 to 0.370, *p* = 0.0036) after three NACT cycles. 

#### 3.2.3. Correlation Analysis between NLR and CRS Score

The ANOVA test showed a significant association between NLR value and CRS (*p* < 0.001); at post hoc analysis, patients with CRS3 showed a significantly lower NLR (mean ± SD: 0.6 ± 0.26) in comparison to patients with CRS 2 (mean ± SD: 1.13 ± 0.17) and 1 (mean ± SD: 1.56 ± 0.8). 

#### 3.2.4. Correlation Analysis between NLR and PFS

**Figure 2:**
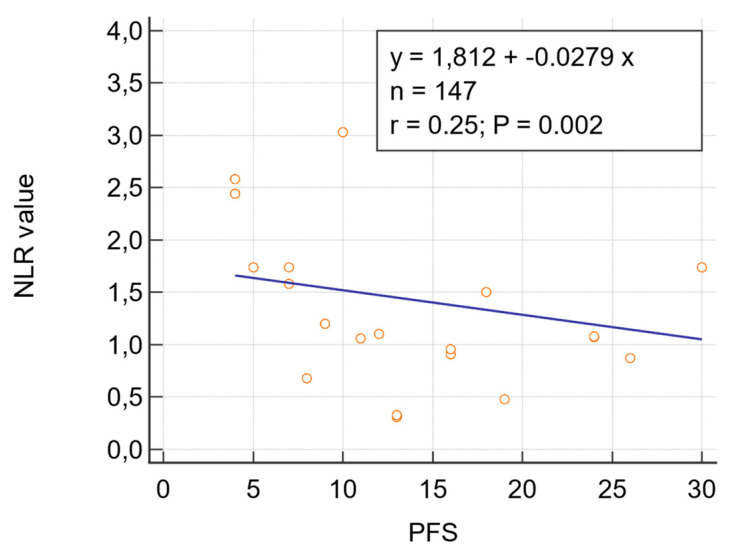
Regression analysis between NLR after three NACT cycles and PFS.

Regression analysis showed a significant inverse association between NLR value after three NACT cycle and PFS (regression coefficient −0.02787; 95% CI: −0.04553 to −0.01021; *p* = 0.0022) (Figure 2). Vice versa, NLR at baseline did not correlate with PFS (regression coefficient −0.1116; 95%CI: −0.3610 to 0.1379; *p* = 0.3609).

#### 3.2.5. ROC Analysis

**Figure 3:**
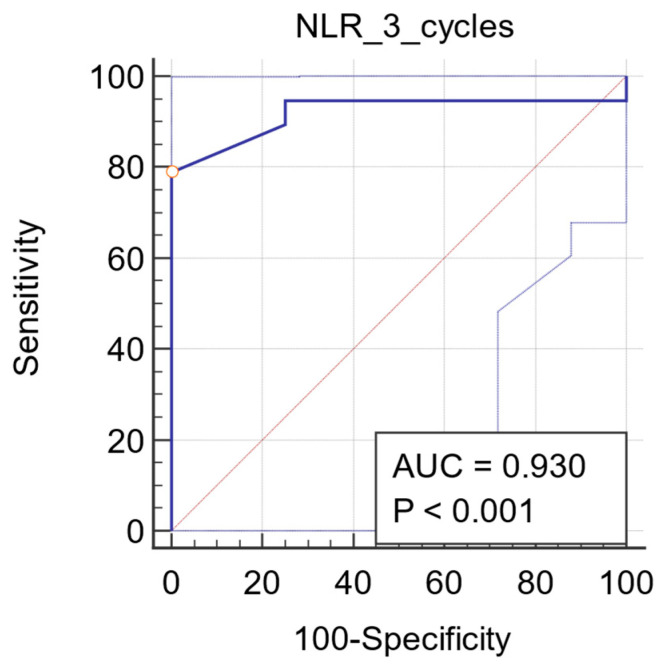
ROC curve for NLR after three cycles. Plot of the receiver operating characteristic curve of the NLR assessed after three cycles of NACT as a predictor of objective tumor response.

**Figure 4:**
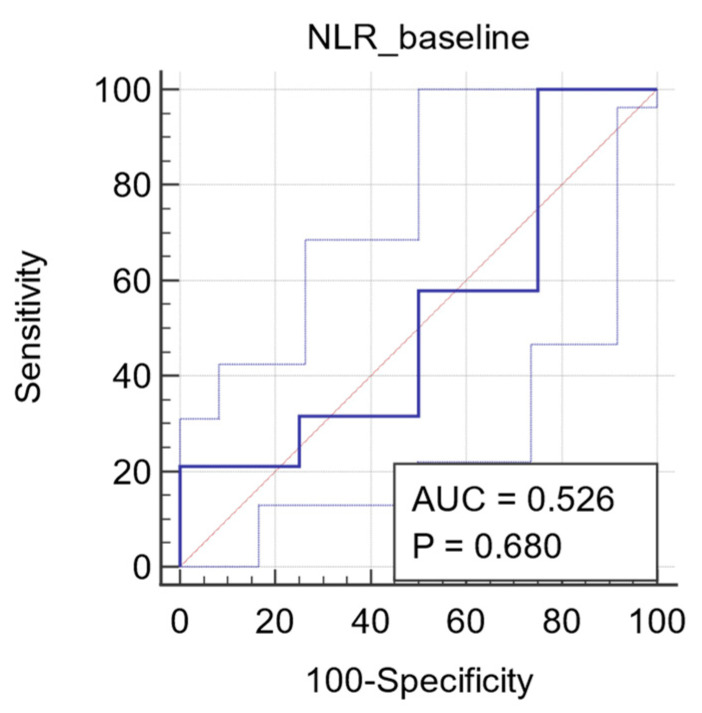
ROC curve for baseline NLR. Plot of the receiver operating characteristic curve of the NLR assessed at baseline as a predictor of objective tumor response.

ROC analysis was used to determine the best cut-off value for NLR as a predictor of objective tumor response. The ROC curve of NLR after three NACT cycles yielded a superior diagnostic accuracy with an AUC of 0.930 (95% CI: 0.868 to 0.958, *p* < 0.0001). The Youden index identified an optimal cutoff of 1.58 with a sensitivity of 79% and a specificity of 100% (Figure 3). Regarding the predictive potential of baseline NLR, the ROC curve only yielded an AUC of 0.526 (95% CI: 0.446 to 0.605, *p* = 0.680; Figure 4).

#### 3.2.6. Kaplan–Meier Curve Analysis of Progression Free Survival by NLR Value after Three Cycles of NACT

**Figure 5:**
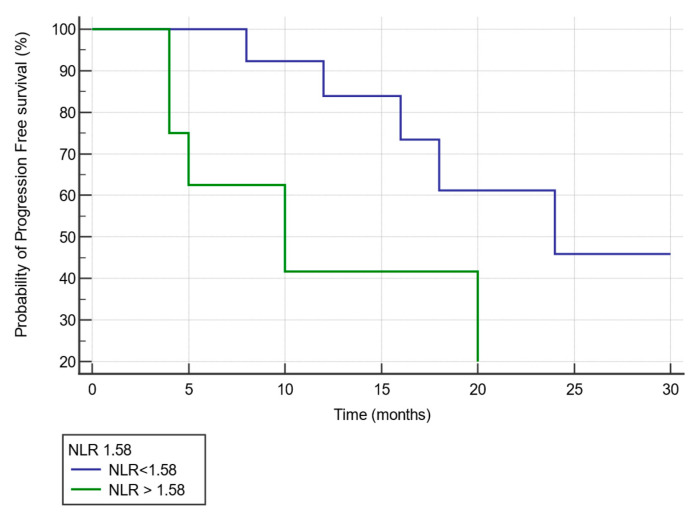
Kaplan–Meier survival curves comparing patients according to NLR value after three cycles of NACT. Patients with a NLR > 1.58 (green line) in comparison to patients with a NLR < 1.58 (blue line) after three cycles of NACT showed a significantly lower progression-free survival (median progression free survival: 10 months versus 24 months, respectively).

We analyzed the difference in PFS by Kaplan–Meier curve analysis and log-rank analysis between patients divided into 2 groups according to the NLR cut off value of 1.58 identified by the ROC curve. We found that patients with an NLR > 1.58 in comparison to those with NLR < 1.58 had a significantly lower PFS (median PFS:10 months versus 24 months, HR 9.3126, 95% CI: 4.9070–17.6734; *p* < 0.0001) (Figure 5).

## 4. Discussion

Inflammation can generate a pro-tumoral microenvironment that can promote ovarian cancer carcinogenesis and aid the growth of the tumors, becoming a systemic condition in the advanced stages [8]. Indeed, the typical symptoms observed in patients with advanced ovarian cancer have been attributed to systemic inflammation and this inflammation is correlated with the outcomes. Nutritional, functional, and immunological decline are the most common inflammation-related symptoms [35].

In the last decades, many authors have investigated the most common indexes of systemic inflammation and their potential application in the stratification of patients with cancer according to the stage of the disease [36]. Therefore, nowadays, inflammation parameters are increasingly becoming promising candidates for the prediction of cancer outcomes [37].

Notably, in recent years, many researchers have investigated the value of blood constituents in the systemic inflammatory response and reported that single elements of a complete blood sample may have clinical utility in the prediction of cancer outcome. Many of these indexes, such as the platelet-to-lymphocyte ratio, monocyte-to-lymphocyte ratio, and, in particular, the NLR are currently used as prognostic indexes [38,39,40].

Regarding the NLR, increasing evidence has shown that neutrophilia is associated with pro-tumoral effects. Neutrophils can assist cancer cell invasion, proliferation, metastasis, and can also participate in the escape of cancer cells from immune surveillance. In contrast, lymphocytes play a fundamental role in tumor defense by inhibiting cell proliferation and migration and inducing cell death [7]. Furthermore, lymphopenia shows the ineffectiveness of the immune surveillance systems, and its association with poor survival in many malignancies, including ovarian cancer, has been reported [41,42].

At present, more than 60 studies (>37,000 patients) have investigated the clinical utility of NLR in the prediction of patient outcomes in a great number of cancers [43].

Several researchers have investigated the potential prognostic role of this index in ovarian cancer. 

Cho et al. [27] were the first authors to describe the effectiveness of NLR as a discriminative biomarker between benign and malignant ovarian masses. They found that pre-operative NLR in combination with CA125 are valid discriminative markers for epithelial ovarian cancer, as increased NLR was observed in malignant disease, but not in benign ovarian tumors. However, in our opinion, the diagnostic criteria of malignancies have been well-established using other parameters; particularly, the ultrasound features of the mass and the presence of high Doppler signals, ascites, and abnormal tumoral markers such as CA125 and HE4 have been validated as diagnostic indexes for ovarian cancer [44,45]. Therefore, the association of NLR with cancer outcomes could be useful in indicating the presence of systemic symptoms that correlate with advanced stages of the disease, but NLR is not necessarily a diagnostic parameter.

Indeed, in a study by Williams et al. [18] a high NLR before treatment was found in association with an advanced FIGO stage, greater tumor grade, and more extensive ascites in patients with ovarian cancer. Furthermore, Miao et al. [28] demonstrated that pre-operative NLR is an independent prognostic marker for the response to adjuvant platinum-based chemotherapy. They retrospectively analyzed a total of 344 patients; the patients were assigned to either a platinum-resistant group or platinum-sensitive group based on their chemotherapeutic responses. Pre-operative NLR <3.02 was associated with a median progression-free survival (PFS) of 33 months; meanwhile, NLR > 3.02 had a median PFS of 11 months, suggesting that a high NLR may be a reliable indicator for chemoresistance.

In the present study, 161 patients with advanced ovarian, fallopian tube, or primary peritoneal HGSC were prospectively enrolled, and the prognostic significance of the NLR was determined in two independent cohorts. We analyzed the correlation between NLR after three cycles of NACT and the objective tumor response. We stratified the patients according to their response to NACT and assessed the correlation to NLR. Of note, we observed that a high baseline NLR was not associated with response to NACT. Conversely, better response to NACT was correlated to a decrease in NLR. Consistently, in patients who underwent interval debulking score we found a statistically significant lower NLR score in patients with CRS3 in comparison to CRS2 and 1. Moreover, we found that patients with a low NLR value after 3 cycles of NACT, but not at baseline, had a significantly higher PFS than patients with high NLR after 3 cycles of NACT. 

Our data are in agreement with the results of Kim at al. [29] who analyzed the role of NLR as a prognostic marker for platinum-based chemotherapy for ovarian cancer by examining NLR changes during chemotherapy. In their study, the authors retrospectively analyzed 203 patients treated by NACT before interval debulking surgery. Pre-treatment NLR was calculated prior to the start of NACT. They defined the modifications of NLR as the post-neoadjuvant NLR value divided by the initial value. The correlation between NLR and dynamic changes during chemotherapy was determined by response score, response rate, and recurrence. They found that a higher pre-treatment NLR (>3.81) was associated with poor overall survival, but not PFS. Dynamic changes in NLR during NACT showed prognostic value for PFS in their cohort. Moreover, they found an association between the increase in NLR during NACT and reduced PFS; in the multivariate analysis, change in NLR was an independent prognostic factor for PFS. Modifications in NLR during chemotherapy were better predictors of PFS than pre-treatment NLR. 

Although many studies have proposed a relationship between a high NLR value and the prognosis of patients with cancer, no clear explanations have been proposed. Various hypotheses have been suggested to elucidate the association between increased NLR and poor outcomes. As previously discussed, the host immune response to tumors is lymphocyte dependent. Patients with increased NLR present a relative lymphocytopenia. Therefore, they may exhibit an impaired lymphocyte-mediated immune response to the tumors, which worsens the prognosis and increases the potential for tumor recurrence in patients. However, this observation is meaningless when evaluated before any treatment. 

We believe that the changes in the number of circulating lymphocytes, and consequently, changes in the NLR, are related to the level of systemic inflammation and to the inflammation-related indexes, and they can be modified by the response to treatment. In fact, our data showed that there is an association between the reduction in CRP, IL-6, and ferritin levels and the increase in circulating lymphocytes.

Therefore, following the lymphocyte trend during chemotherapy can certify and predict response to the treatment. We have recently reviewed how the response to effective surgical and chemotherapy treatment may revert the state of chronic inflammation, thus re-establishing the NLR, and even correcting the condition of neoplastic cachexia that these patients often present with when they are hospitalized before any form of treatment, and which strongly correlates with lymphopenia [13,46]. 

A future prospective is that inflammation markers, including NLR, would be routinely included in clinical practice in ovarian cancer patient evaluation along with validated tumor markers, but they should not be considered prognostic factors at baseline regardless of the stage of disease.

## 5. Conclusions

In conclusion, our results confirmed that the NLR change during treatment could serve as a predictive marker of response to NACT in patients with HGS advanced ovarian cancer. It allows the early identification of non-responsive patients who will need treatment remodeling. Moreover, dynamic changes in NLR during chemotherapy may inform clinicians with an early, more accurate response to treatment of advanced-stage ovarian cancer. 

## Figures and Tables

**Table 1:** Patients’ clinical characteristics.

Characteristics	Mean (Range)
Age (yr)	57 (39–86)
BMI	27 (16–32)
Histology	Mean (%)
HGSC	161 (100.0)
Non-HGSC	0 (0.0)
FIGO stage	Mean (%)
IIIC	47 (29.2)
IVA	76 (47.2)
IVB	38 (23.6)
Performance status	
0–1	30%
2	60%
3	10%
Regimen of NACT	Mean (%)
Taxane + carboplatin	161 (100.0)

Data are shown as median (range) or number (%). Abbreviations: HGSC, high grade serous carcinoma; FIGO, International Federation of Gynecology and Obstetrics; NACT, neoadjuvant chemotherapy.

**Table 2:** Baseline laboratory parameters of the enrolled patients.

	Group 1	Group 2	*p* Value
	N = 132	N = 29	
NLR	4.8 (1.3–13.7)	7.3 (2.7–18.6)	0.6688
CRP (mg/dL)	5.6 (0.1–21)	5.9 (0.4–14.6)	0.0984
Fibrinogen (mg/dL)	556 (318–1052)	598 (299–774)	0.1983
IL-6 (pg/mL)	22.3 ± 8.9	21 ± 1.3	0.2381
Ferritin (ng/mL)	421 ± 147	430 ± 168	0.3876
Albumin (g/dL)	3.1 ± 0.8	3.0 ± 0.7	0.5670
GPS	2 ± 1.5	2 ± 1.8	0.7652

Data are shown as median (range). Groups were compared by Mann–Whitney test for non-parametric variables; *p* < 0.05 was considered statistically significant. Abbreviations: NLR, Neutrophil/Lymphocyte ratio; CRP, C-reactive protein; IL, Interleukin; GPS, Glasgow Prognostic Score.

**Table 3:** Correlation between NLR and other laboratory parameters at baseline and after three cycles of chemotherapy.

	Baseline			After Three Cycles		
	R	*p* Value	CI 95%	r	*p* Value	CI 95%
CRP (mg/L)	0.469	<0.0001 *	0.336 to 0.584	0.422	<0.0001 *	0.438 to 0.541
Fibrinogen	0.089	0.069	−0.335 to 0.484	0.033	0.881	−0.384 to 0.439
Ferritin (ng/mL)	0.329	0.036 *	0.0765 to 0.370	0.554	0.006 *	0.237 to 0.653
IL-6 (pg/mL)	−0.652	0.001 *	0.595 to 0.744	0.600	0.003 *	0.337 to 0.561
Albumin	−0.197	0.083	−0.122 to 0.187	0.235	0.067	−0.0517 to 0.254
GPS	−0.490	0.021 *	0.012 to 0.711	0.390	0.029 *	0.065 to 0.752

* *p* < 0.05 was considered statistically significant. Abbreviations: NLR, Neutrophil/Lymphocyte ratio; CRP, C-reactive protein; IL, Interleukin; GPS, Glasgow Prognostic Score.

**Table 4:** Laboratory parameters of the patients after three cycles of chemotherapy.

	Group 1	Group 2	*p* Value
	N = 132	N = 29	
NLR	1.2 ± 0.8	2.4 ± 0.5	<0.0001
CRP (mg/dL)	0.4 ± 0.3	1.3 ± 1.8	0.0038
Fibrinogen (mg/dL)	373 ± 88	400 ± 114	0.1894
IL-6 (pg/mL)	12.9 ± 10.5	21.3 ± 13.1	0.0068
Ferritin (ng/mL)	351 ± 167	500 ± 268	0.0166
Albumin (g/dL)	3.4 ± 1.1	3.1 ± 0.8	0.0423
GPS	1 ± 0.8	2 ± 1.5	0.0382

Data are shown as median (range). Groups were compared by Mann–Whitney test for non-parametric variables; *p* < 0.05 was considered statistically significant. Abbreviations: NLR, Neutrophil/Lymphocyte ratio; CRP, C-reactive protein; IL, Interleukin; GPS, Glasgow Prognostic Score.

## Data Availability

Original clinical, laboratory and instrumental data can be found in patient charts archived at the Department of Obstetrics and Gynecology and available at request from the corresponding author.
